# Supplementary material for: Structure of the homodimeric androgen receptor ligand-binding domain
Source: Nat Commun. 2017 Feb 6;8:14388. doi: 10.1038/ncomms14388 (PMC5303882; doi:10.1038/ncomms14388)
Supplement: Supplementary Information — Supplementary Figures, Supplementary Tables, Supplementary Notes, Supplementary References. [file ncomms14388-s1.pdf]

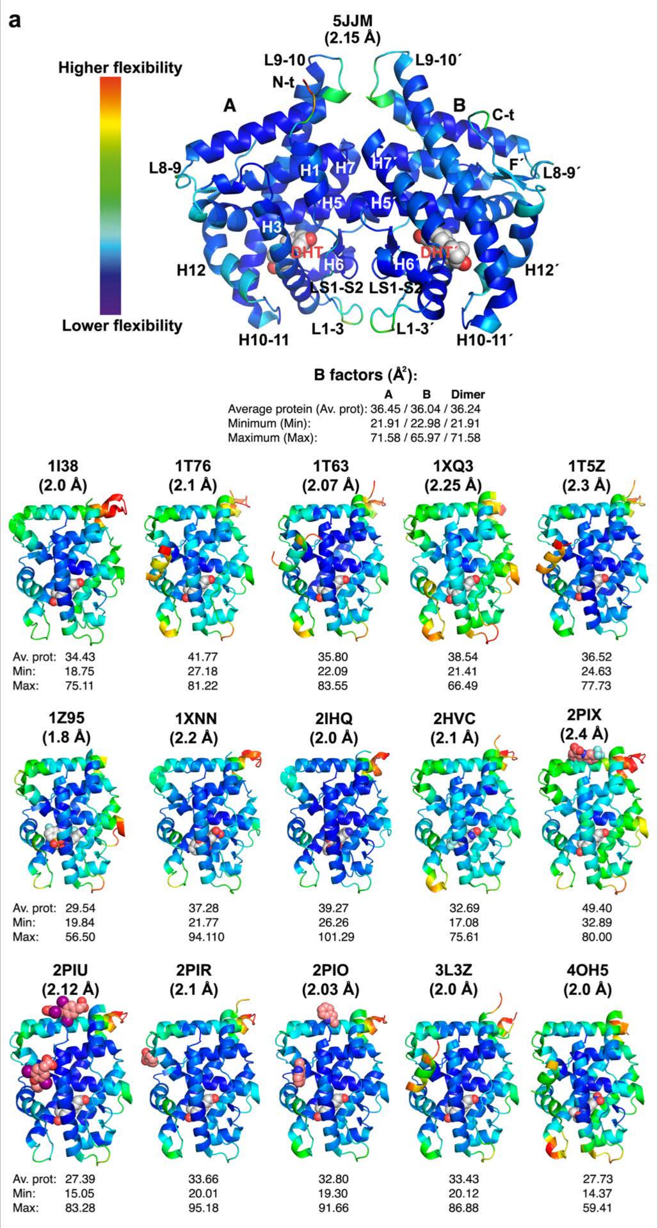
a
5JJM
(2.15 Å)
Higher flexibility
Lower flexibility
L9-10
L9-10´
N-t
A
B
C-t
L8-9
H1
H7
H7´
F´
L8-9´
H5
H5´
H3
DHT
DHT´
H6
H6´
H12
H12´
LS1-S2
LS1-S2´
L1-3
L1-3´
H10-11
H10-11´
B factors (Å²):
A B Dimer
Average protein (Av. prot): 36.45 / 36.04 / 36.24
Minimum (Min): 21.91 / 22.98 / 21.91
Maximum (Max): 71.58 / 65.97 / 71.58
1I38 (2.0 Å)
1T76 (2.1 Å)
1T63 (2.07 Å)
1XQ3 (2.25 Å)
1T5Z (2.3 Å)
Av. prot: 34.43 41.77 35.80 38.54 36.52
Min: 18.75 27.18 22.09 21.41 24.63
Max: 75.11 81.22 83.55 66.49 77.73
1Z95 (1.8 Å)
1XNN (2.2 Å)
2IHQ (2.0 Å)
2HVC (2.1 Å)
2PIX (2.4 Å)
Av. prot: 29.54 37.28 39.27 32.69 49.40
Min: 19.84 21.77 26.26 17.08 32.89
Max: 56.50 94.110 101.29 75.61 80.00
2PIU (2.12 Å)
2PIR (2.1 Å)
2PIO (2.03 Å)
3L3Z (2.0 Å)
4OH5 (2.0 Å)
Av. prot: 27.39 33.66 32.80 33.43 27.73
Min: 15.05 20.01 19.30 20.12 14.37
Max: 83.28 95.18 91.66 86.88 59.41

Supplementary Figure 1 | Comparison of the temperature factors of the AR-LBD in the monomeric and homodimeric crystal forms.

(**a**) The AR-LBD dimer is represented as a cartoon colored according to the average temperature factors (B-factors) of each residue. The 'left' monomer is shown in the standard orientation, i.e. with helix H1 and the AF-2 groove facing the viewer. (**b**) A selection of 15 monomeric AR-LBD structures deposited in the PDB to date and refined to a similar resolution as the current crystal structure. The structures are identified by their PDB codes, and original papers describing these structures are cited in Supplementary References[1-11]. The regions of the protein structure with higher mobility/flexibility are highlighted with warmer colors, those that exhibit lower flexibility/higher stability with colder colors. (B factors were taken as deposited in the corresponding PDB entries, without further manipulations). Notice the overall stabilization of the helical bundle in the AR-LBD homodimer. By contrast, the monomeric form exhibits enhanced flexibility in the L1-3, L9-10 and L4-5 loops, as well as in helix H5, the C-terminal end of H9, and the start of H10.

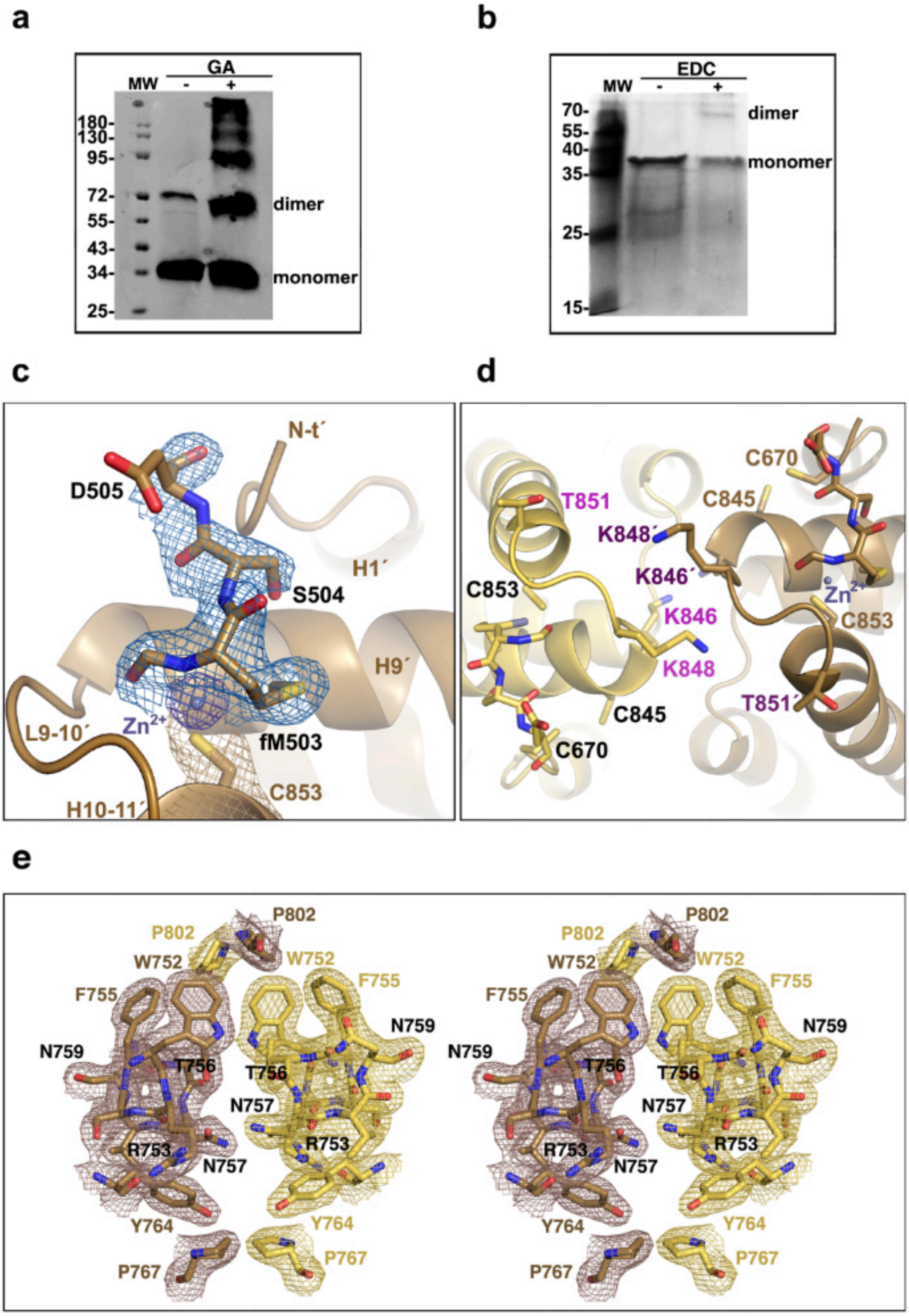
a
MW
GA
-
+
180-
130-
95-
72-
55-
43-
34-
25-
dimer
monomer
b
MW
EDC
-
+
70-
55-
40-
35-
25-
15-
dimer
monomer
c
N-t´
D505
H1´
S504
H9´
L9-10´
Zn2+
fM503
C853
H10-11´
d
C670
T851
C845
K848´
K846´
Zn2+
C853
K846
K848
C845
T851´
C670
e
P802
P802
W752
W752
F755
F755
N759
N759
T756
T756
N757
R753
N757
R753
Y764
Y764
P767
P767

Supplementary Figure 2 | Demonstration of AR-LBD dimer formation in solution and details of the dimer interface.

(**a**) Results of the cross-linking of AR-LBD with glutaraldehyde. A Western blot is shown demonstrating rapid formation of an AR-LBD dimer, along with higher-order multimers. (**b**) Results of the incubation of AR-LBD with the zero-length crosslinker, EDC. Notice appearance of a faint band with a relative molecular mass corresponding to the AR-LBD dimer. (**c**) Closeup of the dimerization interface around residue C853 from monomer B. Notice the presence of extra electron density, which has been tentatively interpreted as a divalent cation coordinated by the Sγ atom, and in addition surrounded by a formylated peptide, probably stemming from recombinant AR-LBD itself. Notice also the close proximity of C687 residues from the two core monomers, B and C. The final electron density map contoured at 1σ is shown. (**d**) Post-translational modifications cluster around the L9-10 loop. Closeup of the AR-LBD dimer interface centered on the L9-10 loops. Note that residues K846 and K848 are known to be ubiquitinated[12], whereas phosphorylation of S792 and T851 has been reported[13]. This is in addition to disulfide bridge formation between cysteine residues at positions 670 and 845 (See ref.[14] and our own unpublished observations). (**e**) Stereo closeup showing major interactions across the interface of the core dimer composed by the arbitrarily labeled molecules B (in yellow) and C (in brown). Electron density is shown as either a brown or yellow mesh contoured at 1σ.

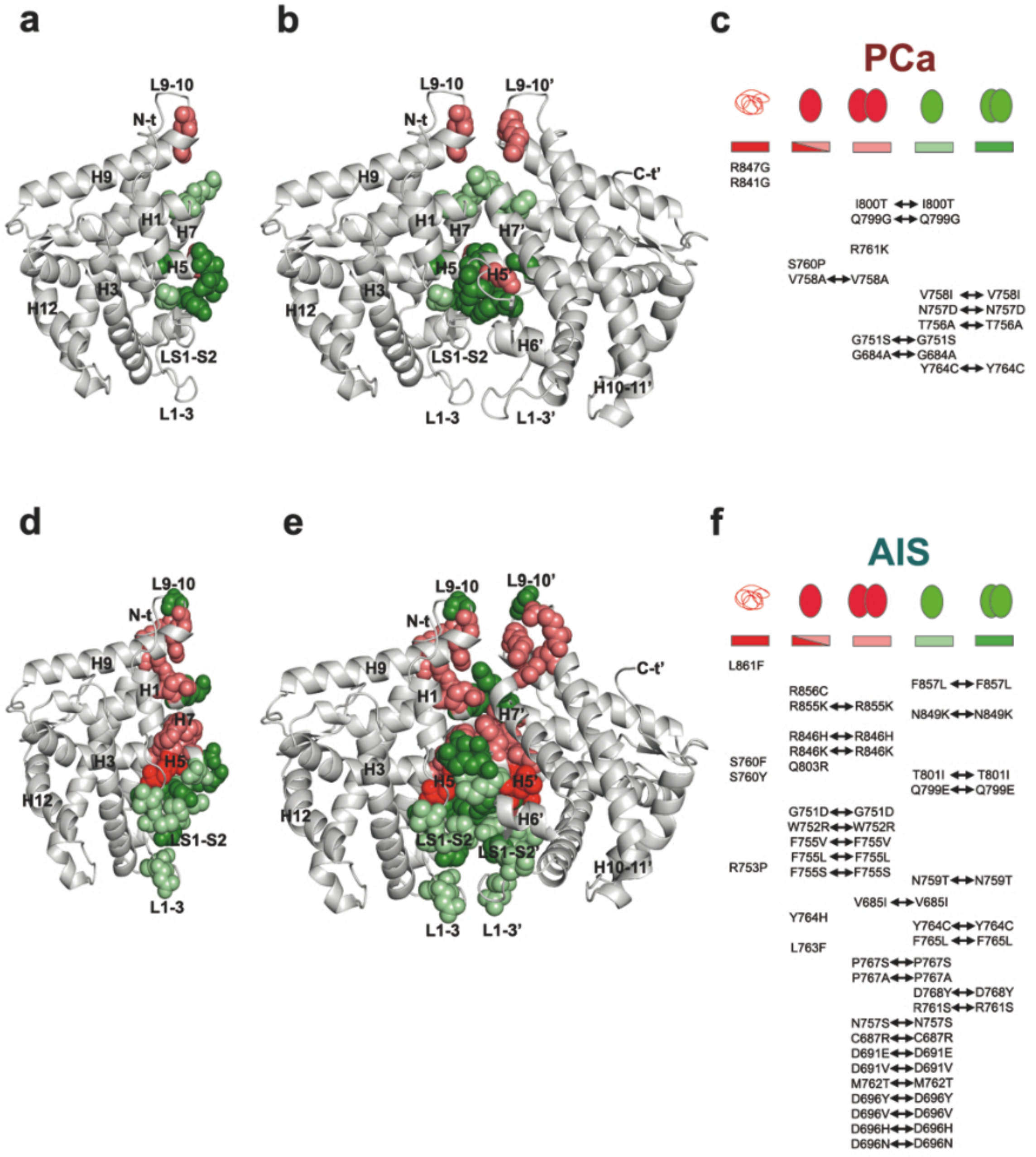
a
L9-10
N-t
H9
H1
H7
H5
H3
H12
LS1-S2
L1-3
b
L9-10
L9-10'
N-t
H9
C-t'
H1
H7
H7'
H5
H5'
H3
H12
H6'
LS1-S2
H10-11'
L1-3
L1-3'
c
PCa
R847G
R841G
I800T ↔ I800T
Q799G ↔ Q799G
R761K
S760P
V758A ↔ V758A
V758I ↔ V758I
N757D ↔ N757D
T756A ↔ T756A
G751S ↔ G751S
G684A ↔ G684A
Y764C ↔ Y764C
d
L9-10
N-t
H9
H1
H7
H3
H5
H12
LS1-S2
L1-3
e
L9-10
L9-10'
N-t
H9
C-t'
H1
H7'
H3
H5
H5'
H12
H6'
LS1-S2
LS1-S2'
H10-11'
L1-3
L1-3'
f
AIS
L861F
F857L ↔ F857L
R856C
R855K ↔ R855K
N849K ↔ N849K
R846H ↔ R846H
R846K ↔ R846K
S760F
S760Y
Q803R
T801I ↔ T801I
Q799E ↔ Q799E
G751D ↔ G751D
W752R ↔ W752R
F755V ↔ F755V
F755L ↔ F755L
R753P
F755S ↔ F755S
N759T ↔ N759T
V685I ↔ V685I
Y764H
Y764C ↔ Y764C
F765L ↔ F765L
L763F
P767S ↔ P767S
P767A ↔ P767A
D768Y ↔ D768Y
R761S ↔ R761S
N757S ↔ N757S
C687R ↔ C687R
D691E ↔ D691E
D691V ↔ D691V
M762T ↔ M762T
D696Y ↔ D696Y
D696V ↔ D696V
D696H ↔ D696H
D696N ↔ D696N

Supplementary Figure 3 | Predicted effects of PCa/AIS-linked mutations on AR-LBD structure and stability.

Monomeric (**a**, **d**) and dimeric (**b**, **e**) AR-LBD structures are represented as cartoons colored light gray. Residues whose mutations have been associated with PCa (**a**, **b**) or AIS (**d**, **e**) are shown with all their non-hydrogen side chain atoms depicted as spheres, colored according to the predicted effect of the exchange on protein folding: dark green (more stable AR-LBD dimer), light green (increased monomer stability), salmon (local structural disorder of the monomer or less active/inactive dimer), or red (local or overall structural disorder of the AR-LBD monomer). Notice that mutations cluster at the dimer interface identified in the current crystal structure. (**c**, **f**) Consensus classification of the predicted effects of PCa or AIS-linked mutations on protein stability obtained with various independent computational tools.

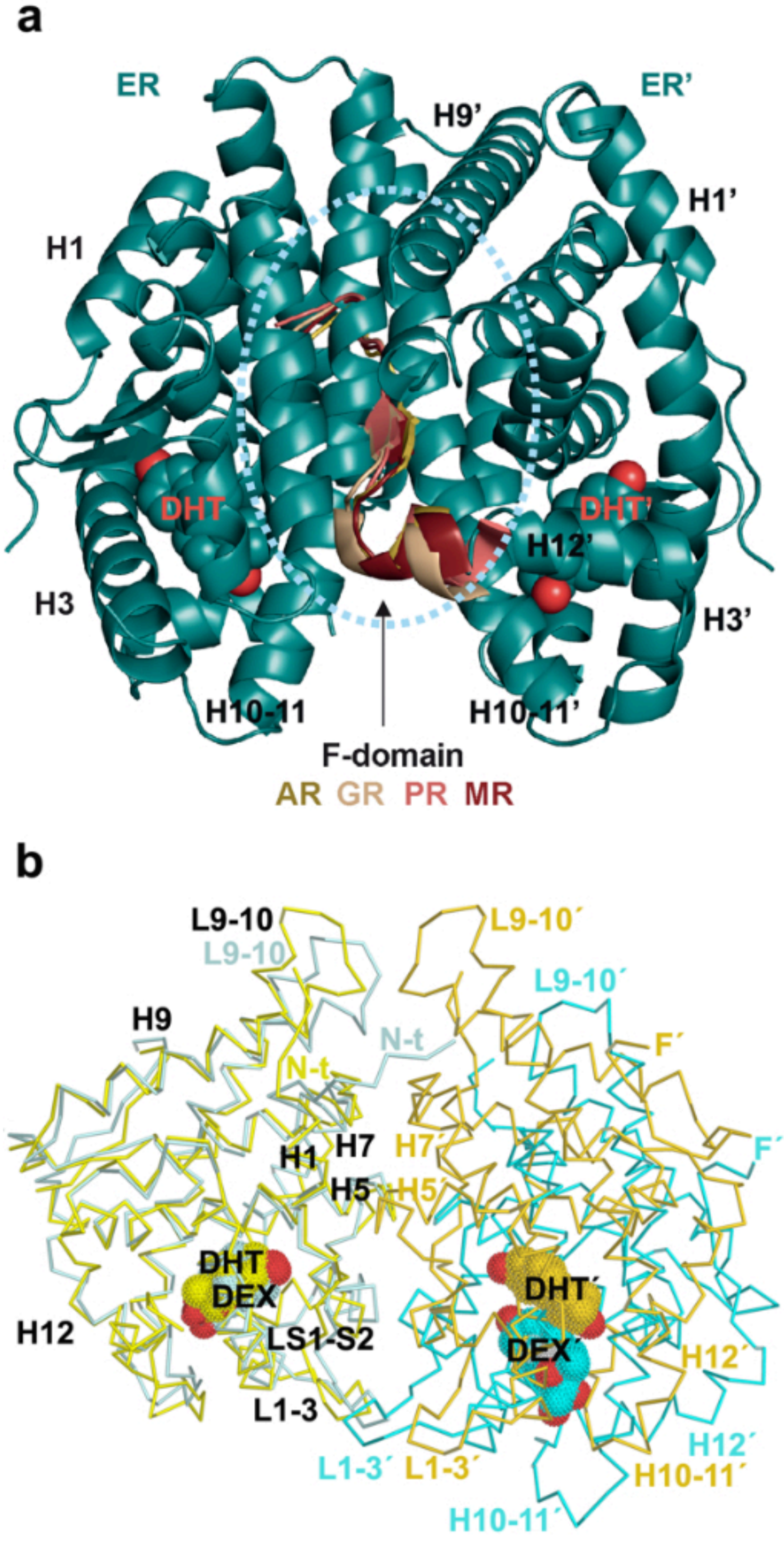
a
ER
ER'
H9'
H1'
H1
DHT
DHT'
H12'
H3
H3'
H10-11
H10-11'
F-domain
AR GR PR MR
b
L9-10
L9-10
L9-10´
L9-10´
H9
N-t
N-t
F´
F´
H1
H7
H7´
H5
H5´
DHT
DEX
DHT´
DEX´
H12
LS1-S2
L1-3
H12´
H12´
L1-3´
L1-3´
H10-11´
H10-11´

Supplementary Figure 4 | Comparison of the current AR-LBD homodimer with previous structures of steroid receptors.

(**a**) Structure of the canonical ERα dimer, shown as a blue cartoon. The C-terminal end of helix H12 and the F-domain of AR (yellow; PDB: 1T7T), GR (light brown; PDB: 1M2Z), PR (salmon; PDB: 1A28) and MR (dark red; 2A3I) are overlaid onto the homodimeric ER structure. Notice that oxosteroid NRs cannot dimerize according to the canonical scheme if the F-domain is wrapped up around the LBD surface. (**b**) Overlay of the Cα traces of AR-LBD and GR-LBD (PDB code 1M2Z) homodimers. The two monomers are shown in yellow and gold and in gray and blue, respectively. Notice that the GR-LBD might easily adopt the symmetric head-to-head conformation seen in the current AR-LBD structure by a simple rotation towards the 2-fold axis (indicated with a curved arrow), accompanied by minor rearrangements of the L9-10 loop and of some interface side chains.

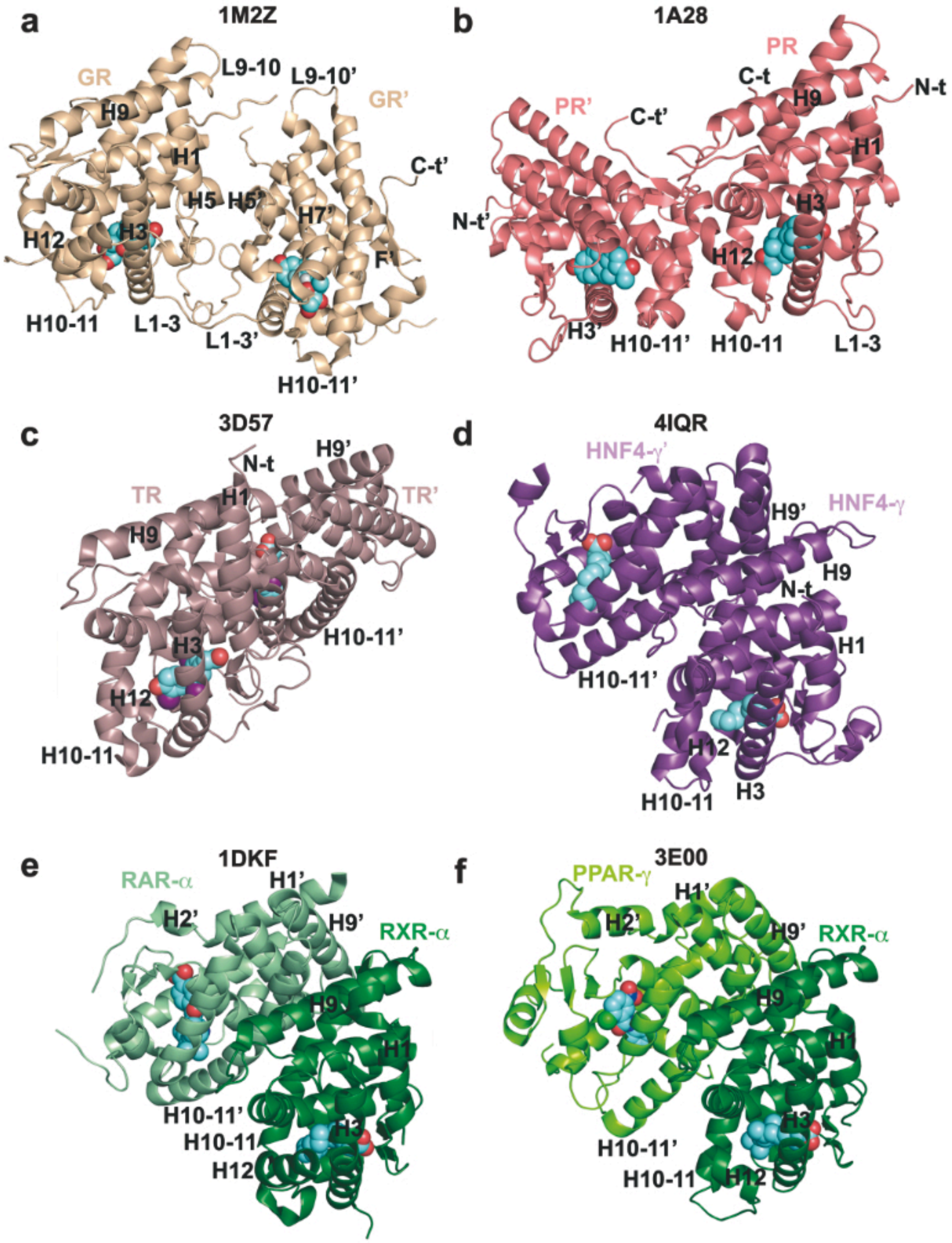
a
1M2Z
GR
L9-10
L9-10'
GR'
H9
H1
C-t'
H5
H5'
H7'
H12
H3
F'
H10-11
L1-3
L1-3'
H10-11'
b
1A28
PR
C-t
N-t
PR'
C-t'
H9
H1
N-t'
H3
H12
H3'
H10-11'
H10-11
L1-3
c
3D57
N-t
H9'
TR
H1
TR'
H9
H10-11'
H3
H12
H10-11
d
4IQR
HNF4-γ'
HNF4-γ
H9'
H9
N-t
H1
H10-11'
H12
H10-11
H3
e
1DKF
RAR-α
H1'
H2'
H9'
RXR-α
H9
H1
H10-11'
H10-11
H3
H12
f
3E00
PPAR-γ
H1'
H2'
H9'
RXR-α
H9
H1
H3
H10-11'
H10-11
H12

Supplementary Figure 5 | Comparison of the AR-LBD with homo- and heterodimers of non-steroid nuclear receptors.

Cartoon representation of the previously reported structures of the dimers of (**a**) GR-LBD (PDB code 1M2Z), (**b**) PR-LBD (1A28; notice that the AF-2 groove would be occluded in this arrangement, casting doubts about its physiological relevance), (**c**) TRβ (3D57), (**d**) HNF4α (4IQR), (**e**) RXRα-RARα (1DKF), and (**f**) PPARγ-RXRα (3E00). The individual domains are colored pale brown (GR), pink (PR), saddle brown (TRβ), purple (HNF4α), forest green (RXRα), olive (RARα), and lawn green (PPARγ). The corresponding ligands are shown as color-coded spheres (carbon, aquamarine blue; red, oxygen). Notice that the TRβ homodimer differs from the canonical dimers shown in panels (d) – (f). The pathological relevance of point mutations that map to the C-terminal end of helix H5 and neighboring areas for generalized thyroid hormone resistance (GTHR) suggests that the TR-LBD homodimer might adopt an AR-like head-to-head conformation in solution.

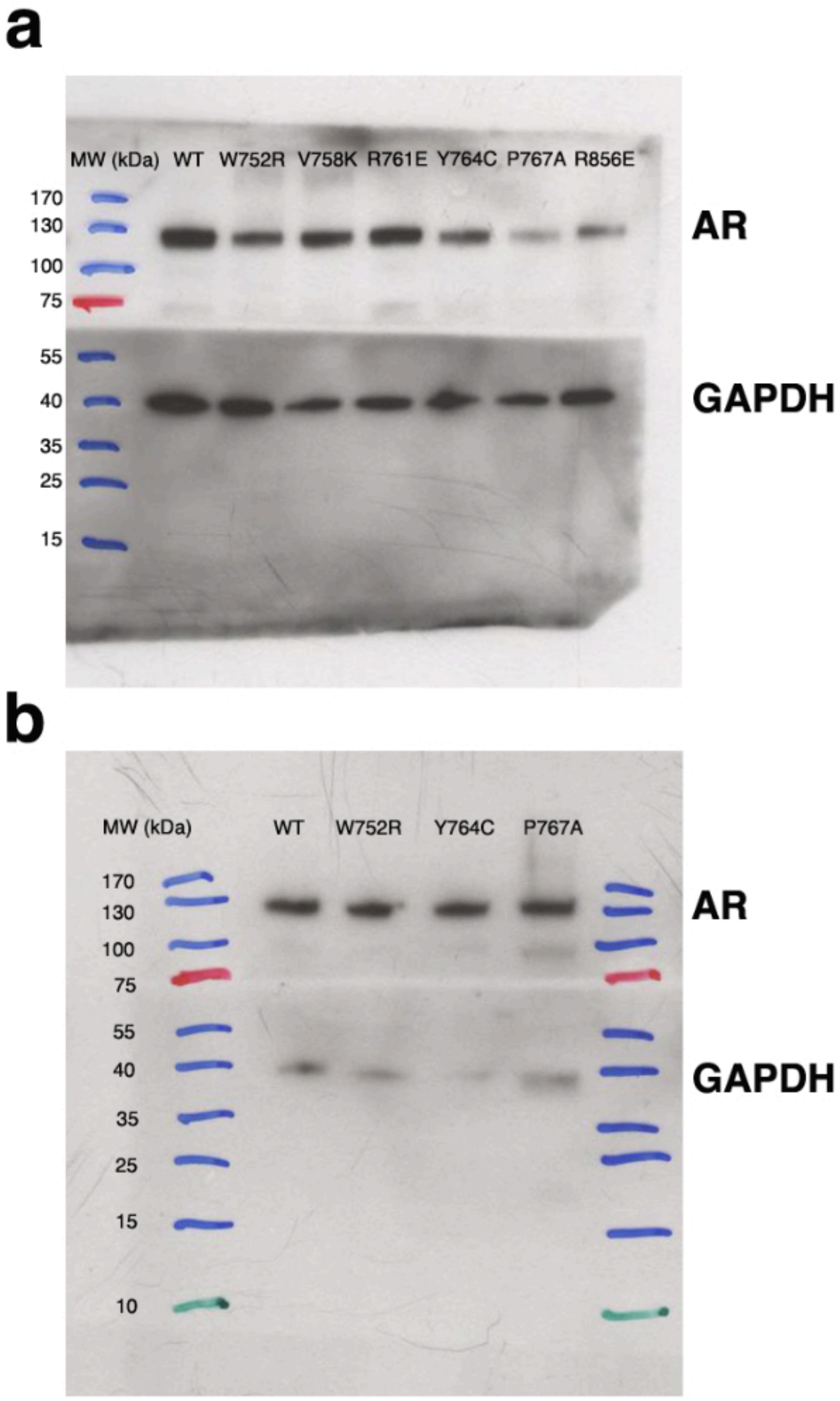
a
MW (kDa)
WT
W752R
V758K
R761E
Y764C
P767A
R856E
170
130
100
75
55
40
35
25
15
AR
GAPDH
b
MW (kDa)
WT
W752R
Y764C
P767A
170
130
100
75
55
40
35
25
15
10
AR
GAPDH

Supplementary Figure 6 | Uncropped scans of the Western blots shown in Figure 5d,e.

Supplementary Tables

Supplementary Table 1 (ST1): Summary of BMOE-crosslinked peptides identified by mass spectrometry.

Peak	m/z	[M+H]+ (exp.)	[M+H]+ (calc.)	δ(ppm)	Peptide 1	Crosslinker	Peptide 2
**BMOE3_6**	1191.034	4761.113	4761.074	8.26	[EAIEPGVVCAGHDNNQPDSF]	BMOE*	[NVLEAIEPGVVCAGHDNNQPDSF]
**BMOE3_7**	1191.033	4761.11	4761.074	7.49	[EAIEPGVVCAGHDNNQPDSF]	BMOE*	[NVLEAIEPGVVCAGHDNNQPDSF]
**BMOE3_8**	1369.148	5473.569	5473.522	8.53	[NVLEAIEPGVVCAGHDNNQPDSF]	BMOE**	[NVLEAIEPGVVCAGHDNNQPDSFAALL]
**BMOE3_10**	1206.048	4821.168	4821.132	7.65	[EAIEPGVVCAGHDNNQPDSF]	BMOE**	[EAIEPGVVCAGHDNNQPDSFAALL]
**BMOE3_11**	1379.661	5515.620	5515.569	9.25	[EAIEPGVVCAGHDNNQPDSFAALL]	BMOE**	[NVLEAIEPGVVCAGHDNNQPDSFAALL]
**BMOE1_15**	1191.032	4761.104	4761.074	6.36	[EAIEPGVVCAGHDNNQPDSF]	BMOE*	[NVLEAIEPGVVCAGHDNNQPDSF]
**BMOE1_17**	1195.533	4779.108	4779.085	4.97	[EAIEPGVVCAGHDNNQPDSF]	BMOE**	[NVLEAIEPGVVCAGHDNNQPDSF]
**BMOE1_18**	1287.597	5147.365	5147.327	7.42	[NVLEAIEPGVVCAGHDNNQPDSF]	BMOE**	[EAIEPGVVCAGHDNNQPDSFAALL]

*Incorporates a water molecule at the basic pH used for chymotrypsinolysis.

**Incorporates two water molecules at the basic pH used for chymotrypsinolysis.

Supplementary Table 2 (ST2): Topologically equivalent interface residues in steroid receptors and in the TRβ.

AR	PR	GR	MR	ER	TR
P850	G862	S709	S914	T496	-
K848	K860	N707	N913	L495	-
K846	R859	R704	C910	A493	K356
R847	Q860	E705	N912	G494	-
R855	S865	Q710	Q916	Q499	H362
R856	R869	R714	R920	R503	F363
Q859	Q872	Q717	Q923	Q506	K366
Q799	Q812	Q657	Q863	N439	N310
T801	S814	S659	T866	Q441	L311
Q803	E816	E661	E867	E443	E315
F755	Y768	Y613	Y819	M396	Y267
N759	S772	S617	N823	P399	P269
V758	V771	S616	T822	H398	-
N757	H770	Q615	H821	H398	-
T756	K769	R614	K820	-	-
P802	Q815	S659	F866	G442	-
W752	W765	W610	W816	W393	V265
R753	R766	R611	R817	R394	R266
M762	M775	L620	F826	K401	T273
Y764	Y777	C622	Y828	L403	T275
P767	P780	P625	P831	P406	E279
C687	Y700	Y545	Y751	F328	-
D768	D781	D626	D832	N406	-
N692	N705	S550	S756	-	D195
N693	T706	S551	S757	-	P193
D691	D704	D549	D755	-	L192
Q694	K707	V552	K758	-	-
P695	P706	P553	P759	F337	-

Supplementary Table 3 (ST3): Bioinformatics analysis of AIS-associated mutations.

AIS	CUPSAT						SDM						PolyPhen program	Istable	Folding RaCe	
	DIMER			MONOMER			DIMER			MONOMER						
Residue	Effect on stability	ΔΔG (Kcal/ mol)	Torsion	Effect on stability	ΔΔG (Kcal/ mol)	Torsion	Effect on stability	ΔΔG (Kcal/ mol)	% Solvent	Effect on stability	ΔΔG (Kcal/ mol)	% Solvent	Score	Score	Dimer	Monomer
V685I	↓	-1,06	Favourable	↑	5,69	Unfavourable	↓	-0,03	18	↑	0,04	77	0,917	73,52	0,79	0,33
C687R	↓	-1,33	Unfavourable	↓	-0,07	Unfavourable	↓	-0,75	54	↓	-0,83		0,997		-1,14	-1,14
D691V	↓	-0,28	Favourable	↑	0,62	Favourable	↑	1,1	68	↑	1,10	71	1	60,37	-1,51	
D691E	↓	-1,9	Unfavourable	↓	-0,54	Unfavourable	↓	0,02	68	↑	0,02	71	0,994	60,37	-1,58	-1,58
D696H	↓	-0,74	Unfavourable	↓	-0,47	Favourable	↑	1,45	23	↑	1,45	21	1	27,06	6,52	6,52
D696N	↑	0,99	Favourable	↑	0,10	Favourable	↓	-0,33	23	↓	-0,33	21	0,999	27,06	-0,03	-0,03
D696Y	↓	-0,55	Unfavourable	↓	-1,37	Unfavourable	↑	1,51	23	↑	1,51	21	1	27,06	1,32	1,32
D696V	↓	-1,05	Unfavourable	↓	-0,30	Unfavourable	↑	0,8	23	↑	0,80	21	0,999	27,06	1,35	1,35
G751D	↑	0,64	Unfavourable	↑	0,64	Unfavourable	↓	-0,35	1,2	↓	-0,34	1,1	1	0,00	5,21	5,21
W752R	↓	-3,24	Favourable	↓	-3,28	Favourable	↓	-2,64	8,2	↓	-2,50	17	1	17,05	-3,57	-2,80
R753Q	↑	1,22	Favourable	↓	-0,10	Unfavourable	↓	-1,33	15,4	↓	-0,45	30	1	25,76	-0,17	-0,71
R753P				↑	0,17	Unfavourable	↓	-3,44	15,4	↓	-2,28	30	1	25,76	1,65	1,65
F755V	↓	-1,02	Favourable	↓	-0,86	Favourable	↓	-0,48	11	↓	-0,33	29	0,994	24,90	-0,30	-2,39
F755S	↓	-2,81	Unfavourable	↓	-1,83	Favourable	↓	-4,3	11	↓	-3,05	29	1	24,90	-1,89	-1,55
F755L	↓	-2,38	Favourable	↓	-1,86	Favourable	↑	0,34	11	↑	0,29	29	0,971	24,90	-0,18	-0,02
N757S	↓	-1,33	Favourable	↑	0,32	Unfavourable	↓	-0,58	23	↓	-0,58	41	0,97	75,14	-0,47	-2,10
N759T	↓	-1,12	Unfavourable	↓	-1,27	Unfavourable	↓	-2,36	22	↑	0,09	85	0,981	5,97	-1,13	-0,50
S760F	↓	-4,43	Unfavourable	↓	-0,13	Unfavourable	↑	1,51	5	↑	1,53	4	1	5,97	-1,99	-1,99
S760Y	↓	-7,73	Unfavourable	↓	-0,93	Unfavourable	↑	0,76	5	↑	1,34	4	1	5,97	-3,83	-3,83
R761S	↑	0,68	Unfavourable	↓	1,38	Favourable	↓	-1,68	35	↓	-0,06	67	0,994	67,69	1,28	-0,50
M762T	↑	1,26	Favourable	↑	2,59	Favourable	↓	-0,63	37	↓	-0,63	33	0,795	25,50	-0,26	-0,26
L763F	↓	-2,61	Favourable	↓	-2,39	Unfavourable	↓	-0,47	0,1	↑	0,45	14	1	0,55	-0,18	-0,18
Y764H	↓	-4,4	Favourable	↑	0,89	Favourable	↓	-2,29	4	↓	-0,97	19	1	21,50	-1,81	-4,15
Y764C	↑	1,94	Favourable	↓	-0,30	Favourable	↑	1,65	4	↑	2,78	19	1	21,53	1,27	-3,25
F765L	↑	2,06	Favourable	↑	1,97	Favourable	↓	-0,28	11,1	↓	-0,55	0	1	9,47		
P767S	↓	-0,49	Unfavourable	↓	0,89	Unfavourable	↓	-1,1	32	↓	-0,49	32	1	40,80	-0,47	-1,48
P767A	↓	-2,8	Favourable	↓	-1,27	Favourable	↑	0,3	32	↑	0,44	32	1	40,80	0,29	-1,24
D768Y	↑	1,06	Unfavourable	↓	0,03	Favourable	↑	1,88	23	↓	-0,22	31	1	20,82	-2,81	-2,81
Q799E	↓	-1,19	Unfavourable	↓	-0,69	Unfavourable	↓	-1,66	40	↓	-0,88	96		87,35	-0,66	-0,66
Q803R	↓	-0,4	Favourable	↑	0,25	Favourable	↓	-0,26	11	↓	-0,26	44	0,986	36,95	-0,04	-3,73
R841C	↑	2,82	Unfavourable	↓	-1,56	Unfavourable	↑	1,11	18	↑	1,11	53	1	53,28		
R841H	↑	1,24	Unfavourable	↑	0,01	Unfavourable	↑	0,4	18	↑	0,40	53	1	53,28		
R841S	↑	0,18	Unfavourable	↑	0,39	Unfavourable	↓	-1,54	18	↓	-1,58	53	1	53,28		
R841G	↓	-0,27	Unfavourable	↓	-1,50	Unfavourable	↓	-1,54	18	↓	-3,25	53	1			
R855K							↓	-0,23	68	↓	-0,23	71			-4,61	-4,61
R856C	↓	-0,41	Unfavourable	↓	-1,72	Unfavourable	↑	0,47	8	↓	-1,86	8	1	4,80	2,72	2,72
R856H	↓	-1,64	Unfavourable	↓	-0,97	Unfavourable	↓	-0,67	8	↓	-2,12	8	1	4,80	14,08	14,08

Supplementary Table 4 (ST4): Bioinformatics analysis of PCa-linked mutations.

PCa	CUPSAT						SDM								PolyPhen program	Folding RaCe	
	DIMER			MONOMER			DIMER				MONOMER						
Residue	Effect on stability	ΔΔG (Kcal/mol)	Torsion	Effect on stability	ΔΔG (Kcal/mol)	Torsion	Effect on stability	ΔΔG (Kcal/mol)	% Solvent	Disruption	Effect on stability	ΔΔG (Kcal/mol)	% Solvent	Disruption	Score	Dimer	Monomer
G684A	↓	-2,60	Unfavourable	↑	2,48	Unfavourable	↑	2,23	30	No-H – No-H	↑	2,23	115	No-H – No-H	0,916	0,04	-1,09
G751S	↓	-0,14	Unfavourable	↑	0,74	Unfavourable	-	0,13	1,2	No-H - Sat	-	0,13	1	No-H - Sat	0,988	6,2	6,2
F755L	↓	-2,38	Favourable	↓	-3,37	Favourable	-	0,29	10	No-H – No-H	-	0,29	29	No-H – No-H	0,971	-0,18	-0,02
T756A	↓	-0,18	Favourable	↑	1,07	Favourable	↑	2,21	7	Sat – No-H	↑	1,97	48	Sat – No-H	0,196	0,48	-0,92
N757D	↑	0,79	Favourable	↑	1,44	Unfavourable	↓	-0,68	10	No-H – No-H	↓	-0,63	41	Sat - Sat	0,99	0,37	-1,45
V758A	↓	-2,15	Unfavourable	↓	-1,41	Unfavourable	-	0,23	4	No-H – No-H	-	-0,68	14	No-H – No-H	0,536	-1,04	-1,04
V758I	↑	0,81	Unfavourable	↓	-0,24	Unfavourable	↑	0,59	4	No-H – No-H	↑	0,53	14	No-H – No-H	0,965	0,72	0,72
S760P	↓	-5,45	Unfavourable	↓	-3,12	Unfavourable	↓	-8,87	5	Sat – No-H	↓	-8,72	4	Sat – No-H	0,999	-4,34	-4,34
R761K	↓	-0,95	Favourable	↓	-0,26	Favourable	-	0,3	35	No-H – No-H	-	0,09	67	Sat - Sat	0,047		
Y764C	↑	1,94	Favourable	↓	-0,3	Favourable	↑	2,78	4	No-H – No-H	↑	2,11	19	No-H - Sat	1	1,27	-3,25
Q799G	↓	-1,46	Favourable	↓	-1,76	Unfavourable	↑	0,77	40	No-H – No-H	↑	0,77	96	No-H – No-H	0,998	0,29	0,29
I800T	↓	-10,7	Unfavourable	↑	2,42	Unfavourable	↓	-2,06	0	No-H - Sat	↓	-3,08	0	UnSat – No-H	0,999	-1,78	-1,78
R847G	↓	-1,00	Unfavourable	↓	-0,5	Unfavourable	↓	-3,02	25	Sat – No-H	↓	-2,61	28	Sat – No-H	0,986	-0,11	-0,11

Supplementary Table 5 (ST5): Functional data reported on PCa- and AIS-associated mutations clustering at the dimer interface.

Residue	Phenotype	Ligand binding	Transactivation	Reference
G684A	PCa		↓	Shi et al., 2002; Qi et al., 2013
V685I	CAIS	Bmax zero		Mebarki et al., 1993
C687R	PAIS			Hiort et al., 1996
D691E	PAIS	WT	↓	Hughes et al., 2012
D691V	CAIS	KD for DHT is 4.5x > WT	↓	Tadokoro et al., 2009
D696H	CAIS	Bmax low		Ris-Stalpers et al., 1991
D696N	MAIS, PAIS, CAIS	WT	↓	Ris-Stalpers et al., 1991; Jääskeläinen et al., 2006; Helsen et al., 2012
D696V	CAIS			Dork et al., 1998
D696Y	CAIS			Audi et al., 2010
G751D	CAIS	Bmax very low	↓	Bevan et al., 1997
G751S	PCa		WT	Shi et al., 2002
W752R	PCa, CAIS	Low to normal Bmax, KD > WT		Langley et al., 1998; Boehmer et al. 2001
R753P	CAIS			Hughes et al., 2012
R753Q	CAIS	KD for R1881 is 1.5x > WT. Dissociation time is 12x > WT	↓	Langley et al., 1998
F755L	PCa, PAIS	KD for DHT is 1.5x > WT	↓	Shi et al., 2002; Tadokoro et al., 2009
F755S	MAIS, PAIS	KD for DHT is 3x > WT	↓	Tadokoro et al., 2009
F755V	CAIS	KD for DHT is 5x > WT	↓	Tadokoro et al., 2009
T756A	PCa		↓	Hay and McEwan, 2012
N757D	PCa		↓	Hay and McEwan, 2012
N757S	MAIS, PAIS	KD for DHT is > WT	↓	Giwercman et al., 2001
V758A	PCa		WT	Shi et al., 2002; Hay and McEwan, 2012; Qi et al., 2013
V758I	PCa		↓	Hay and McEwan, 2012
N759T	PAIS	WT	↓	Yong et al., 1998; Helsen et al., 2012
S760F	CAIS	Bmax zero		DeBellis et al., 1992
S760P	PCa		↓	Shi et al., 2002; Hay and McEwan, 2012
S760Y	CAIS			Hughes et al., 2012
R761S	PAIS		↑	Helsen et al., 2012
M762T	PAIS			Ledig et al., 2005
L763F	CAIS	No binding		Bevan et al., 1997
Y764C	PCa, PAIS	Normal KD/High KD. Dissociation half life (3H)R1881 = 35% of WT.	↓	Murono et al., 1995; Jääskeläinen et al., 2006; Hay and McEwan, 2012; Langley et al., 1998
Y764H	CAIS	KD is > WT. Dissociation half life (3H)R1881 = 23% of WT.	↓	Langley et al., 1998
F765L	CAIS	Bmax low. Abnormal dissociation rate.	↓	Szafran et al., 2009; Marcelli et al., 1994
P767A	CAIS	WT	↓	Helsen et al., 2012
P767S	CAIS			Hay and McEwan, 2012
D768E	CAIS	Bmax very low		Lobaccaro et al., 1993; Melo et al., 1998
D768Y	CAIS			Taes et al., 2009
Q799E	PCa, MAIS, PAIS	WT	WT/↓	Castagnaro et al., 1993; Shi et al., 2002; Jääskeläinen et al., 2006; Hay and McEwan, 2012; Qi et al., 2013
I800T	PCa			Sanchez et al., 2006
Q803R	PAIS			Chavez et al., 2001
R841C	PAIS	KD for R1881 is 3x > WT	↓	Georget et al., 1998
R841G	PAIS	Bmax low	↓	Giwercman et al., 1998
R841H	PAIS, CAIS	Bmax low, KD for DHT is 15x > WT		Marcelli et al., 1994
R841S	PAIS			Mazen et al., 2004
R847G	PCa		WT	Shi et al., 2002
R855K	PAIS	Bmax low		McPhaul et al., 1992
R856C	CAIS	No binding	Inactive	Murono et al., 1995; Elhaji et al., 2004
R856H	MAIS, PAIS, CAIS	Normal Bmax and dissociation rate, KD for R1881 is 2x > WT	↓	Elhaji et al., 2004; Jääskeläinen et al., 2006

Supplementary Notes

The major implications of the current structure for disease-associated point mutations are summarized below.

Mutations associated with androgen insensitivity syndromes:

In addition to the large number of AIS-linked mutations that cluster at the core of the AR-LBD dimer interface, some variants affect the "upper" L5-6 and L7-8 loops (N759T, S760F/Y, R761S, M762T, Y764C/H, P767S/A and Q799E) or the lower area of the interface (V685I, C687R, D691V/E, D696N/Y/V/L and D768Y) (Figures 5a,c, and Supplementary Tables 3,5). Several of these AIS-associated mutations may lead to local structural disorder of the AR monomer, which might explain their pathogenicity (e.g. W752R, R753P, S760F/Y, L763F and R856C) (Supplementary Figure 3).

Most reported replacements, however, affect residues that are well exposed in the AR-LBD monomer and are predicted to be neutral or even favorable for its structure, but are involved in important inter-monomer contacts, and would therefore compromise the stability of the dimer (Supplementary Figure 3 and Supplementary Table 3). Mutations F755L and F755V, in particular, have an interesting correlation with AIS severity: the Phe→Leu exchange is associated with a less severe PAIS phenotype, while introduction of the related aliphatic residue, Val, results in CAIS[15] (see also Supplementary Table 5). Although both leucine and valine would be equally well tolerated at this position in monomeric AR-LBD, inspection of the current structure reveals that a Leu side chain could still engage in vdW interactions with residue P802 from the adjacent monomer, albeit weaker than those formed by the bulkier wild-type Phe (Figure 2d). In contrast, a smaller Val side chain cannot participate in such interactions, and therefore the F755V mutant is predicted to remain in a monomeric state, disrupting biological activities of the receptor and explaining the complete female phenotype of carriers of this mutation. In a similar manner, loss of important H-bonds across the dimer axis would compromise dimer formation and/or stability (Figure 2e), which explains the deleterious impact of replacing residue R761 by Ser (reported in PAIS patients) and of the N757 exchange to Ser (MAIS/PAIS). Finally, impaired vdW interactions across the dimer interface (Figure 2d) are likely to underlie the CAIS phenotype in carriers of the P767A/S mutations. Indeed, we could demonstrate that the P767A variant interferes with dimer formation, in spite of retained ligand binding ability (see Figure 4c and main text for details).

Mutations associated with metastatic prostate cancer:

Some of these AR variants (in particular R847G, but also S760P and R841G) are predicted to destabilize the LBD monomer, while mutations that cluster in the central part of the interface are more preservative of structural integrity and ligand binding: G684S, G751S, Q799G, and I800T are predicted to stabilize the monomer. On the other hand, PCa-associated mutations that affect residues exposed on H5 (F755L, T756A, N757D and V758A/I), or neighboring areas (R761K, Y764C) are likely to be neutral regarding the structure of the monomer (Figures 5b,c, Supplementary Figure 3, and Supplementary Tables 4-5). For the exposed structure-preserving mutations T756A, N757D, V758I and Y764C, all bioinformatics tools predict enhanced dimerization (Supplementary Table 4). In the cases of T756A and Y764C the smaller size of the mutant side chains might facilitate a tighter dimer assembly. On the other hand, the V758I exchange is predicted to stabilize the AR-LBD dimer thanks to additional vdW interactions of the bulkier I758 side chain with residues M763 and Y764 from the neighboring monomer. Interestingly, mutation Y764C strongly enhanced the activity of the AR in our functional studies (Figure 5), which is in accordance with the general belief that overactive AR can lead to therapy resistance in advanced PCa[16].

Supplementary References

1 Sack, J. S. *et al.* Crystallographic structures of the ligand-binding domains of the androgen receptor and its T877A mutant complexed with the natural agonist dihydrotestosterone. *Proc. Natl. Acad. Sci. U.S.A.* **98**, 4904-4909 (2001).

2 Hur, E. *et al.* Recognition and accommodation at the androgen receptor coactivator binding interface. *PLoS Biol.* **2**, e274 (2004).

3 Estébanez-Perpiñá, E. *et al.* The molecular mechanisms of coactivator utilization in ligand-dependent transactivation by the androgen receptor. *J. Biol. Chem.* **280**, 8060-8068 (2005).

4 He, B. *et al.* Structural basis for androgen receptor interdomain and coactivator interactions suggests a transition in nuclear receptor activation function dominance. *Mol. Cell* **16**, 425-438 (2004).

5 Bohl, C. E., Gao, W., Miller, D. D., Bell, C. E. & Dalton, J. T. Structural basis for antagonism and resistance of bicalutamide in prostate cancer. *Proc. Natl. Acad. Sci. U. S. A.* **102**, 6201-6206 (2005).

6 Salvati, M. E. *et al.* Structure based approach to the design of bicyclic-1H-isoindole-1,3(2H)-dione based androgen receptor antagonists. *Bioorg. Med. Chem. Lett.* **15**, 271-276 (2005).

7 Sun, C. *et al.* Discovery of potent, orally-active, and muscle-selective androgen receptor modulators based on an *N*-aryl-hydroxybicyclohydantoin scaffold. *J. Med. Chem.* **49**, 7596-7599 (2006).

8 Wang, F. *et al.* Structure of the ligand-binding domain (LBD) of human androgen receptor in complex with a selective modulator LGD2226. *Acta Crystallogr. Sect. F Struct. Biol. Cryst. Commun.* **62**, 1067-1071 (2006).

9 Estébanez-Perpiñá, E. *et al.* A surface on the androgen receptor that allosterically regulates coactivator binding. *Proc. Natl. Acad. Sci. U.S.A.* **104**, 16074-16079 (2007).

10 Zhou, X. E. *et al.* Identification of SRC3/AIB1 as a preferred coactivator for Hhrmone-activated androgen receptor. *J. Biol. Chem.* **285**, 9161-9171 (2010).

11 Hsu, C.-L. *et al.* Identification of a new androgen receptor (AR) co-regulator BUD31 and related peptides to suppress wild-type and mutated AR-mediated prostate cancer growth via peptide screening and X-ray structure analysis. *Mol. Oncol.* **8**, 1575-1587 (2014).

12 Xu, K. *et al.* Regulation of androgen receptor transcriptional activity and specificity by RNF6-induced ubiquitination. *Cancer Cell* **15**, 270-282 (2009).

13 Lin, H.-K., Yeh, S., Kang, H.-Y. & Chang, C. Akt suppresses androgen-induced apoptosis by phosphorylating and inhibiting androgen receptor. *Proc. Natl. Acad. Sci. U.S.A.* **98**, 7200-7205 (2001).

14 Matias, P. M. *et al.* Structural evidence for ligand specificity in the binding domain of the human androgen receptor: Implications for pathogenic gene mutations *J. Biol. Chem.* **275**, 26164-26171 (2000).

15 Tadokoro, R., Bunch, T., Schwabe, J. W. R., Hughes, I. A. & Murphy, J. C. Comparison of the molecular consequences of different mutations at residue 754 and 690 of the androgen receptor (AR) and androgen insensitivity syndrome (AIS) phenotype. *Clin. Endocrinol.* **71**, 253-260 (2009).

16 Attard, G. *et al.* Prostate cancer. *Lancet* **387**, 70-82 (2016).
